# Microalgae-bacteria symbiosis enhanced nitrogen removal from wastewater in an inversed fluidized bed bioreactor: performance and microflora

**DOI:** 10.3389/fmicb.2025.1591974

**Published:** 2025-04-28

**Authors:** Xin Zheng, Ruoting Liu, Kai Li, Junhao Sun, Kanming Wang, Yuanyuan Shao, Zhongce Hu, Jesse Zhu, Zhiyan Pan, George Nakhla

**Affiliations:** ^1^College of Environment, Zhejiang University of Technology, Hangzhou, China; ^2^Department of Chemical and Biochemical Engineering, The University of Western Ontario, London, ON, Canada; ^3^Wenzhou Ecological and Environmental Monitoring Center of Zhejiang Province, Wenzhou, China; ^4^Nottingham Ningbo China Beacons of Excellence Research and Innovation Institute, The University of Nottingham Ningbo China, Ningbo, China; ^5^College of Biotechnology and Bioengineering, Zhejiang University of Technology, Hangzhou, China

**Keywords:** microalgal-bacterial symbiosis system, biological nitrogen removal, mass balance, simultaneous nitrification and denitrification, syntrophic microbial communities

## Abstract

Conventional wastewater biological nitrogen removal (BNR) processes require a large amount of air and external organic carbon, causing a significant increase in operating costs and potential secondary pollution. Herein, this study investigated the nitrogen removal performance and the underlying mechanisms of a novel simultaneous nitrification and denitrification (SND) coupled with photoautotrophic assimilation system in an inversed fluidized bed bioreactor (IFBBR). Nitrogen removal was achieved through the synergistic interaction of microalgae and bacteria, with microalgae providing O_2_ for nitrification and microbial biomass decay supplying organic carbon for denitrification. The IFBBR was continuously operated for more than 240 days without aeration and external organic carbon, the total nitrogen (TN) removal efficiency reached over 95%. A novel C-N-O dynamic balance model was constructed, revealing that nitrification and denitrification were the primary pathways for nitrogen removal. The model further quantified the microbial contributions, showing that microalgae generated O_2_ at a rate of 81.82 mg/L·d, while microbial biomass decay released organic carbon at a rate of 148.66 mg/L·d. Microbial diversity analysis confirmed the majority presence of microalgae (*Trebouxiophyceae*), nitrifying bacteria (*Gordonia* and *Nitrosomonas*) and denitrifying bacteria (*Ignavibacterium* and *Limnobacter*). This study successfully achieved enhanced nitrogen removal without the need for aeration or external organic carbon. These advancements provide valuable insights into efficient wastewater nitrogen removal, offering significant benefits in terms of reduced energy consumption, lower operational costs, and decreased CO_2_ emissions.

## 1 Introduction

Nitrogen is one of the essential nutrients for the growth of organisms in the water. However, excess nitrogenous compounds discharged into natural water can result in water quality deterioration and eutrophication (Gu et al., 2024). Biological nitrogen removal (BNR) process has been widely used for removing the nitrogen compounds from municipal wastewater (Maheepala et al., 2023; Wang et al., 2022). However, conventional BNR processes separate the nitrification and denitrification processes due to their different redox condition requirements (Alzate Marin et al., 2016; Ye et al., 2021), leading to large footprints (Manser et al., 2016), potential emission of greenhouse gases like N_2_O (Tariq et al., 2025), high aeration (Yan et al., 2024), and external organic matter (Peng et al., 2020).

Recently, many researchers have focused on novel BNR technologies to enhance the nutrient removal efficiency (Wang et al., 2020; Zheng et al., 2024). Fluidized bed bioreactor (FBBR) has the merits of better mixing, enhanced mass transfer (Chowdhury and Nakhla, 2022; Nelson et al., 2017), higher microbial concentrations and activity, greater resistance to impact loads, and less residual biosolids produced (Wang et al., 2020). However, the high flow rate required by the FBBR to achieve fluidization causes high energy consumption, and high shear forces which lead to biofilm detachment (Andalib et al., 2012). The inverse fluidized bed bioreactor (IFBBR), designed with carrier particles slightly less dense than water, requires significantly less fluidization energy and minimizes shear forces. This system has been successfully used to cultivate nitrifying and denitrifying biofilms with synthetic wastewater (Wang et al., 2020; Zhang et al., 2020). With the formation and thickening of biofilm in the IFBBR, the outer and inner layers of biofilm can achieve aerobic conditions and anaerobic/anoxic conditions, respectively, thus facilitating the occurrence of simultaneous nitrification and denitrification (SND). As a novel BNR process, SND incorporates diverse metabolic pathways that could combine aerobic nitrification and anaerobic/anoxic/aerobic denitrification in one reactor (Luan et al., 2023), relying on the cooperation of nitrifiers and denitrifiers (Jin et al., 2023). However, the SND process still requires high aeration for nitrification and external organic carbon for denitrification, which significantly increases energy consumption and operational costs. Moreover, maintaining stable and harmonious cooperation between nitrification and denitrification remains a challenge, limiting its practical potential (Zhang F. et al., 2021). Therefore, if an endogenous supply of oxygen and organic carbon can be established within the SND process, enabling nitrification without aeration and denitrification without external organic carbon, as well as maintaining high nitrogen removal efficiency. Such a BNR process would offer significant potential for further research and practical applications.

Microalgal-bacterial symbiosis systems for municipal wastewater treatment, especially for nitrogen removal, have recently gained great attention, as they can greatly reduce energy, chemicals consumption and carbon dioxide release (Li et al., 2023; Lu et al., 2022). In the microalgal-bacterial symbiosis system, microalgae capture carbon dioxide released by bacteria or dissolved in water and then produce oxygen for bacterial metabolism and growth through photosynthesis (Abouhend et al., 2018; Aparicio et al., 2024), which can save 40–60% of the total energy demand in wastewater treatment (Gu et al., 2021; Huang et al., 2022). Meanwhile, organic carbon synthesized through photosynthesis could be an electron donor demand for denitrifiers (Wang et al., 2015). Tang et al. (2018) suggested that microalgae positively affected nitrogen removal in BNR process in two ways: preferential uptake of ammonia for biomass synthesis (direct effect) and increasing the activity of bacteria by synergism (indirect effect). Moreover, studies have indicated that microalgae may affect the composition and characteristics of extracellular polymeric substances (EPS) and increase floc size, facilitating the formation of anoxic/aerobic microenvironments in one system (Jin et al., 2023).

However, the effects of microalgae on the SND process, and the interactions between microalgae and bacteria, which combine diverse nitrogen removal pathways for collaboration, have yet to be thoroughly elucidated. Once the interaction mechanisms between microalgae and various functional bacteria in the SND denitrification system are clarified, efforts can focus on achieving a low-energy, low-cost, and efficient BNR process without the need for aeration or external organic carbon. This goal requires a comprehensive understanding of the microbial community structure, the migration and transformation pathways of elements, and the specific contributions of functional microbial communities at each stage. Dynamic analysis of C-N-O elements is particularly critical, as the complexity of the process makes it impossible to directly track each element. Researchers are exploring simulation models to address this challenge. However, existing models are typically designed for systems with simple elements or microbial compositions and hard to perform adequately in complex BNR systems.

This study aimed to enhance nitrogen removal by coupling SND with photoautotrophic assimilation in the IFBBR, which was fed with synthetic wastewater, started up and operated for 240 days. In this system, oxygen and organic carbon required for SND can be generated *in situ* by microalgae, eliminating the need for aeration or external organic carbon. Different nitrogen loading rates (NLR) were tested. Microbial community analysis was further conducted to detect the presence and abundance of microalgae and bacteria within the system. In addition, changes in alkalinity, which often accompany migration and transformation of nitrogen but are overlooked, were utilized to construct a novel C-N-O dynamic balance model. This model was used to identify the primary mechanisms for nitrogen removal in the IFBBR. In summary, this study successfully realized the enhanced nitrogen removal from wastewater by coupling SND with photoautotrophic assimilation in an IFBBR and successfully eliminated aeration and organic carbon needs, while the construction and application of a C-N-O dynamic balance model provided valuable insights for nitrogen removal mechanisms, offering an efficient solution for BNR process with reduced energy consumption, chemical usage, and carbon emissions.

## 2 Materials and methods

### 2.1 Inoculum and synthetic wastewater composition

Thickened waste activated sludge (TWAS) was initially collected from wastewater treatment plants and continuously operated in a laboratory-scale Anammox sequencing batch reactor (SBR) for over 300 days, demonstrating a stable nitrogen removal performance in the SBR without aeration and external organic carbon. However, the nitrogen removal rate (NRR) was only 0.02 kg N/m^3^·d with the NLR of 0.26 kg N/m^3^·d. To enhance the nitrogen removal performance, 150 mL of SBR biosolids (mixed liquor volatile suspended solids concentration (MLVSS) of 920 mg/L) was inoculated into the IFBBR.

The composition of synthetic wastewater fed into the IFBBR was kept consistent with that of the Anammox SBR, primarily consisting of sodium nitrite and ammonium sulfate, as shown in Table 1, with KHCO_3_ as the alkalinity source (500 mg/L). The influent ammonia nitrogen concentrations were kept at roughly 58–66 mg/L, roughly double the typical concentrations in municipal wastewater, while the influent nitrite nitrogen varied from 77 to 90 mg/L (Table 1) and some nitrite nitrogen was oxidated into nitrate nitrogen during dissolution. The other constituents of the synthetic medium are listed in Supplementary Table 1 of the Supplementary material. The initial pH was adjusted to about 7.8 using either 0.1 M HCl or 0.1 M NaOH. To avoid interference from external oxygen on the system, synthetic wastewater was first purged with nitrogen gas (>99%) for 30 min and then sealed by a nitrogen gas balloon before being fed into the IFBBR.

**Table 1:** Operating conditions of each phase.

Phase	pH	HRT	Inf. NH_4_^+^-N	Inf. NO_2_^−^-N	Inf. NO_3_^−^-N	NLR
Phase I	7.8–8.0	48 h	58 ± 3 mg/L	77 ± 4 mg/L	10 ± 3 mg/L	0.07 kg N/m^3^·d
Phase II	7.8–8.0	48 h	58 ± 3 mg/L	77 ± 4 mg/L	10 ± 3 mg/L	0.07 kg N/m^3^·d
Phase III	7.8–8.2	48 h	66 ± 6 mg/L	90 ± 5 mg/L	22 ± 4 mg/L	0.09 kg N/m^3^·d

### 2.2 Experimental setup and operating conditions

**Figure 1:**
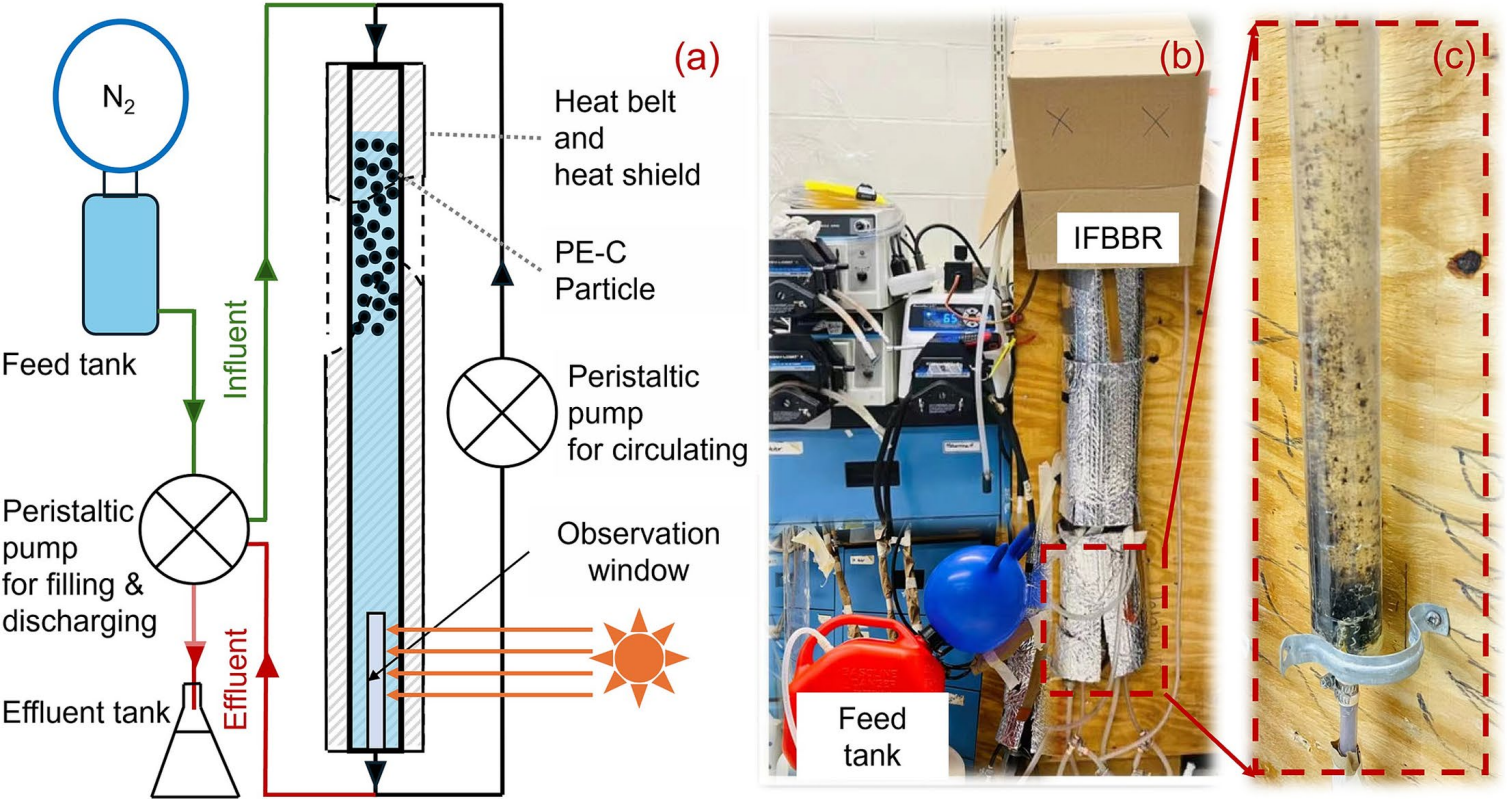
Schematic diagram **(a)**, actual **(b)** of IFBBR and biofilm at the bottom wall of IFBBR after operation of 190 days **(c)**.

An IFBBR was developed in this study to enhance the nitrogen removal performance (Figure 1). The working volume of the polymethyl methacrylate (PMMA) bioreactor was approximately 500 mL with an inner diameter of 3 cm and a height of 100 cm. A heat belt (HTWC 101–010, Omegalux, USA) and heat shield were mounted on the outer layer to keep the temperature at approximately 38°C, which was identical to the temperature in the Anammox SBR. An observation window with an area of 15 square centimeters was set to observe whether the carrier particles were blocked at the bottom. Polyethylene coated carbon (PEC) particles (diameter of 2.0 mm, density of 950 kg/m^3^, roundness of 0.97, nonporous with specific surface area of approximately 14.94 cm^2^/g) were used as carrier particles in the IFBBR with a packing ratio of 30%. After being ultrasonically cleaned three times, there was no detectable total organic carbon in the cleaning water, confirming that the PEC particles could not provide organic carbon to the system. Unlike conventional FBBRs, the PEC particles in this IFBBR have a slightly lower density than water, allowing them to float easily and achieve fluidization with significantly lower water flow. This design reduces the reactor’s energy consumption for fluidization while minimizing shear forces, creating an optimal environment for biofilm growth.

The reactor was operated in 4 h cycles with each cycle including filling & discharging (10 min) and reaction (230 min). The reactor was manually purged with nitrogen gas once daily to reduce the dissolved oxygen concentration (DO) to avoid interference from external oxygen. The pH value of the IFBBR was manually detected at regular intervals. The light intensity in the laboratory is 2,500 lx, and the lights were turned on for approximately 12 h every day.

The particle fluidization was realized by circulating water from the bottom to the top using peristaltic pump (07528–10, Masterflex, USA) at a reflux ratio of 125: 1 based on the influent flow rate. Synthetic wastewater was pumped into the top and discharged from the bottom of the IFBBR through another peristaltic pump (77521–50, Masterflex, USA).

The whole experiment lasted 240 days and was divided into three phases (Table 1): phase I (day 1–28) was the start-up period, Phase II (day 29–87) was the lower nitrogen loading rate (NLR, 0.07 kg N/m^3^·d) period, and phase III (day 88–240) was the higher NLR period, with the NLR increased to 0.09 kg N/m^3^·d.

### 2.3 Batch tests

In order to test the activity of nitritation (AOB), nitratation (NOB) and denitrification (denitrifier), in-situ batch tests were carried out at the end of phase III when total nitrogen (TN) removal efficiency was over 90% and the temperature of 38°C. The composition and concentration of initial solutions were the same as the daily feeding except for the nitrogen compounds (NH_4_^+^-N, NO_3_^−^-N, NO_2_^−^-N). In the three batch tests, (NH_4_)_2_SO_4_, NaNO_2_ and NaNO_3_ were used as the only nitrogen compounds at N concentrations of 35 mg/L, 90 mg/L, and 60 mg/L, respectively. The microbial activity of nitrifiers and denitrifiers in the IFBBR was determined by the specific nitrogen removal rate (SNRR), which was calculated by the slope of the trend of ammonia, nitrite and nitrate concentration with time, respectively. Before each stage, liquid in the IFBBR was drained into a 500 mL Erlenmeyer flask. Subsequently, the initial solutions were slowly added to the IFBBR. The recirculation pump was turned on until the carrier particles floated naturally. Samples were collected at regular time intervals. After each sample collection, the system was purged with nitrogen gas for 15 min to avoid interference from external oxygen. The IFBBR ran normally for 1 to 2 days between each batch test to ensure that its performance was not affected.

### 2.4 Analytical methods

Liquid samples were collected at regular intervals to measure total COD (tCOD), total suspended solids (TSS) and volatile suspended solids (VSS). Soluble samples were passed through 0.45 μm filter membranes (VWR 28333–1390, China) for measuring NH_4_^+^-N, NO_2_^−^-N, NO_3_^−^-N, TN and soluble COD (sCOD) concentrations using an ultraviolet–visible spectrophotometer (DR 3900, Hach Company, USA) according to standard methods (APHA, 2012). An alkalinity auto-titrator was used for the measurement of pH and alkalinity (Alk.) (285212831, Schott, Germany), and Δalkalinity (ΔAlk.) was calculated as the effluent Alk. minus the influent Alk.

### 2.5 Microbial community analysis

On day 190 when TN removal efficiency was over 90%, samples were collected for the microbial community analysis. As the biofilm grew on both the surface and the bottom wall of the IFBBR, the microbial community of biofilm on the carrier and the IFBBR wall was analyzed by IRDA lab (Quebec, QC, Canada). Illumina MiSeq 2 × 300 bp sequencing was performed by the Genomic Analysis Platform of the Institute for Integrative Biology and Systems (IBIS) at Laval University (Quebec, QC, Canada). Sample preparation and DNA extraction were conducted by collecting 2.0 mL homogeneous volume of each sample using the FastDNA Spin Kit for Soil Extraction Kit (MP Biomedicals, Solon, OH, USA) (Gu et al., 2024). The quality and quantity of the extracted genomic DNA samples were determined spectrophotometrically with absorbance measurements at 260 and 280 nm using an A260/A280 ratio. Amplification of the V4 and V6 regions of archaea, bacteria 16S rRNA and eukaryote 18S rRNA were performed using the primer sequences of the specific regions (Apprill et al., 2015). A two-step dual-indexed PCR approach specifically designed for the Illumina MiSeq sequencing platform was performed. The amplicon libraries were sequenced in the paired-end format with a reading of 300 bases, 2 × 300 base pairs on each side of the DNA strand on Illumina MiSeq at the genomic analysis platform, IBIS of University Laval (Quebec, Canada). Bacteria and microalgae with the relative abundance of over 0.7% were analyzed.

### 2.6 C-N-O dynamic balance models

Based on the microbial community analysis and the nitrogen removal theory of microalgae and bacteria, a C-N-O dynamic balance model was developed to further ensure the nitrogen removal mechanisms.

In this microalgal-bacterial symbiosis system, NH_4_^+^-N was removed through two primary pathways: nitrification and microalgae assimilation (Zhang H. et al., 2021). NO_2_^−^-N was generated via nitritation (AOB) and denitratation (DN_3_), while it was removed by nitratation (NOB) and denitritation (DN_2_). NO_3_^−^-N was generated by nitratation (NOB) and removed by denitratation (DN_3_). Organic carbon was generated from biomass decay and then consumed by denitrification. Oxygen, produced by microalgae, was primary utilized by nitrifying bacteria. Combined with the dynamic changes in alkalinity observed during the nitrogen removal in the IFBBR, the C-N-O dynamic balance model was constructed. Detailed derivation of the model is provided in Supplementary Text 1, with the results of C-N-O mass balance presented in section 3.4.

## 3 Results and discussion

### 3.1 Nitrogen removal performance of the IFBBR

**Figure 2:**
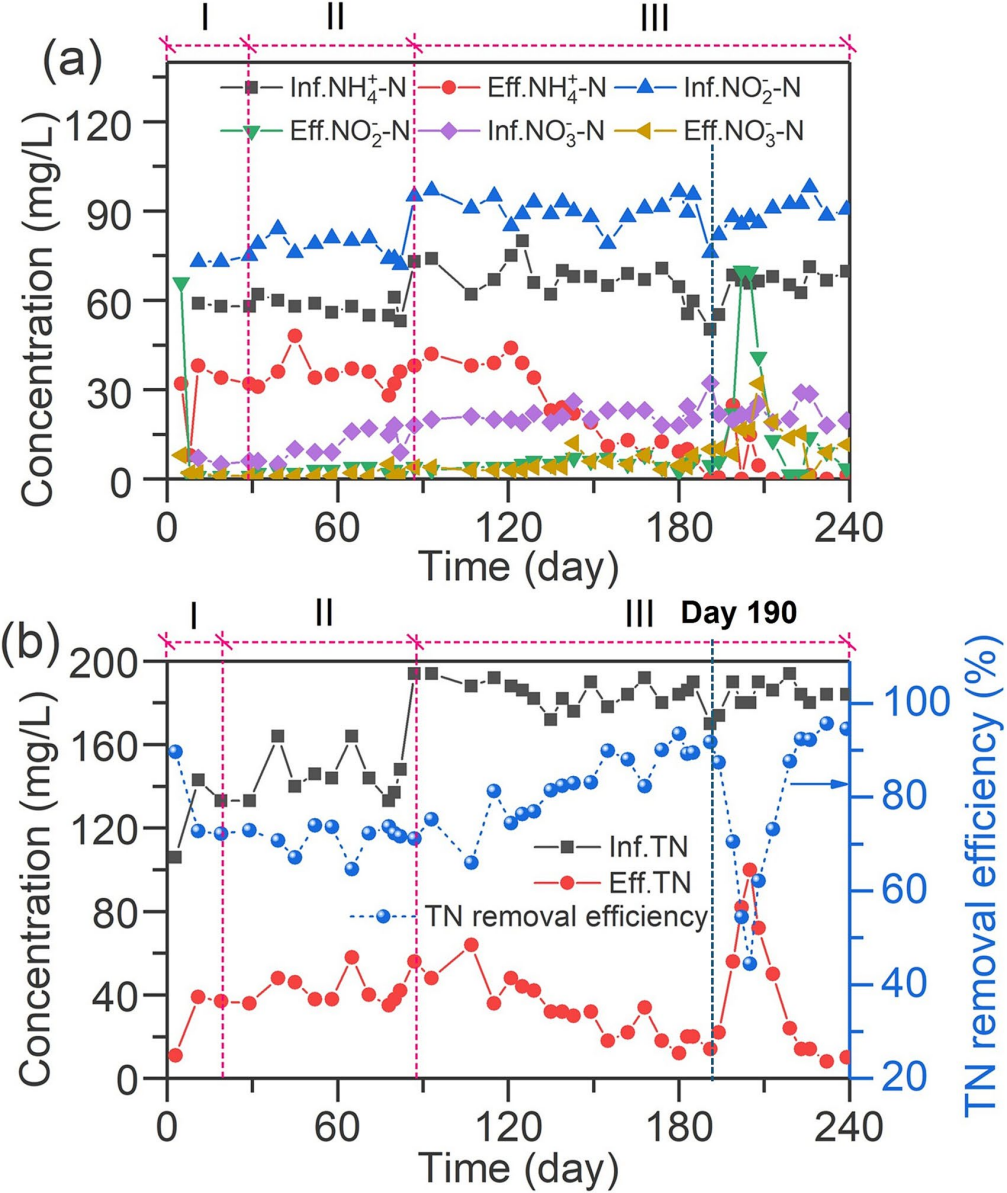
Nitrogen removal performance of the Inversed Fluidized Bed Bioreactor (IFBBR). **(a)** Temporal variations of inorganic nitrogen (NH_4_^+^-N, NO_2_^−^-N, NO_3_^−^-N); **(b)** Temporal variations of total nitrogen removal efficiency.

The temporal variations of NH_4_^+^-N, NO_2_^−^-N, NO_3_^−^-N concentrations in the IFBBR during the experiment are shown in Figure 2a. In phase I, it is obvious that the concentrations of NH_4_^+^-N, NO_2_^−^-N, NO_3_^−^-N in the effluent showed a sharp decrease, then increased and remained stable after day 11. The sharp decrease can be due to the microorganisms consuming nitrogen compounds during the initial period of internal circulation without feeding. Additionally, the concentration of nitrogen compounds in effluent remained stable in the subsequent period indicating the successful start-up of the IFBBR. Figure 2b shows the TN removal performance in the IFBBR, in which the TN removal efficiency remained stable at 72 ± 2% during the remaining time of Phase I (day 1–28), which also indicates the successful start-up of the IFBBR. Interestingly, the seed biosolids was collected from the previous SBR, which had been continuously worked for more than 300 days with the TN removal efficiency between 10 and 20%, NLR of 0.26 kg N/m^3^·d and nitrogen removal rate (NRR) of 0.02 kg N/m^3^·d. However, once the seed biosolids were inoculated into the IFBBR, the biofilm in the IFBBR had a stable and high nitrogen removal performance rapidly. This could be due to the low shear force of the IFBBR and the rough surface and large specific surface area of PEC particles, resulting in suitable conditions for the biofilm formation and the rapid start-up of the BNR process.

During phases I and II (days 29–87) with a low NLR of 0.07 kg N/m^3^·d, the effluent NH_4_^+^-N concentration remained stable at 35 mg/L, while NO_2_^−^-N remained below 10 mg/L. This result indicated that stable nitrogen removal in the IFBBR was achieved. In phase III (day 88–240), NLR was increased to 0.09 kg N/m^3^·d to promote the growth and activity of the biofilm, leading to about a month of water quality fluctuations. It is interesting to point out that the effluent NH_4_^+^ -N concentration showed a significant decreasing trend after 121 days operation and was undetected (less than 1 mg/L) after 190 days operation. It has been widely reported that ammonia nitrogen was usually consumed by nitrification (Svehla et al., 2023), while microalgae also had the capability of ammonia assimilation (Yan et al., 2022), indicating a significant growth of nitrifiers or microalgae in the IFBBR during this period. Meanwhile, the effluent nitrite and nitrate nitrogen concentrations remained below 10 mg/L, which were much lower than the consumption of ammonia, with the TN removal efficiency increasing and reaching over 90% and the NRR of 0.08 kg N/m^3^·d, indicating that the denitrification process occurred simultaneously with the nitrification in the IFBBR. Between day 190 and day 205, due to the collection of biological samples for microbial community analysis, the TN removal efficiency significantly decreased and dropped to 44%. Surprisingly, in the following 15 days, the TN removal efficiency quickly recovered and reached over 95%, further demonstrating the stability of the nitrogen removal performance of the IFBBR.

Nitrifiers require a significant amount of oxygen for their growth and metabolism. However, the feed tank and the IFBBR were purged daily with nitrogen gas and sealed with parafilm. Additionally, the growth and metabolism of denitrifiers require organic carbon, but the only carbon source in the nutrient solution was HCO_3_^−^. These may be due to the presence of microalgae, which produces oxygen and organic carbon for SND. The seed sludge used in the previous SBR was the TWAS collected at the Greenway wastewater treatment plant in London, Canada, which could contain indigenous microalgae (Huang et al., 2022). In the long-term operation of the IFBBR, weak light entered through the observation window, leading to microalgae growing and accumulating at the bottom of the IFBBR. As shown in Figure 1 (day 190), there is indeed a biofilm-like microalgae growing on the wall, microbial community analysis in section 3.3 further proves the presence of microalgae in the IFBBR. In summary, SND and microalgal photoautotrophic assimilation were coupled in the IFBBR. Microalgae provide the necessary oxygen and organic carbon source for SND, while bacteria supply CO_2_ to microalgae. It is precisely due to the presence of microalgae that enhanced the nitrogen removal performance, especially the ammonia removal efficiency, significantly improved after day 121.

In conventional microalgal-bacterial symbiosis systems, it is difficult for microalgae to supply sufficient oxygen and organic carbon to support bacterial growth and metabolism. As a result, additional conditions such as strong light sources, aeration, and external organic carbon supplementation are often required to sustain bacterial activity and ensure the system’s stable operation. Jin et al. (2023) integrated microalgae with simultaneous nitrification and denitrification in microalgal-bacterial sequencing batch reactors (MB-SBR) illuminated by a sunlight-simulating light source. With aeration and the addition of organic carbon sources (starch and glucose), the MB-SBR achieved a TN removal efficiency of 66.74%. Bucci et al. (2024) developed an algal-bacterial granular system in an SBR for the aerobic treatment of cheese whey wastewaters. The system was continuously illuminated using light-emitting diodes (LED) and exhibited COD, ammonia and inorganic nitrogen removal rates of 100, 94, and 30%, respectively, without aeration. Li et al. (2023) investigated nitrogen removal in six enclosed, open and aerated reactors. Under oxygen-limited and glucose-sufficient conditions, with a 12-h light phase (5,000 ± 500 lux) followed by a 12-h dark phase, the algal-bacterial consortium achieved enhanced TN removal of 74.6%. However, in this study, without aeration and external organic carbon, TN removal efficiency of 95% was achieved in the IFBBR with the illumination of weak light.

**Figure 3:**
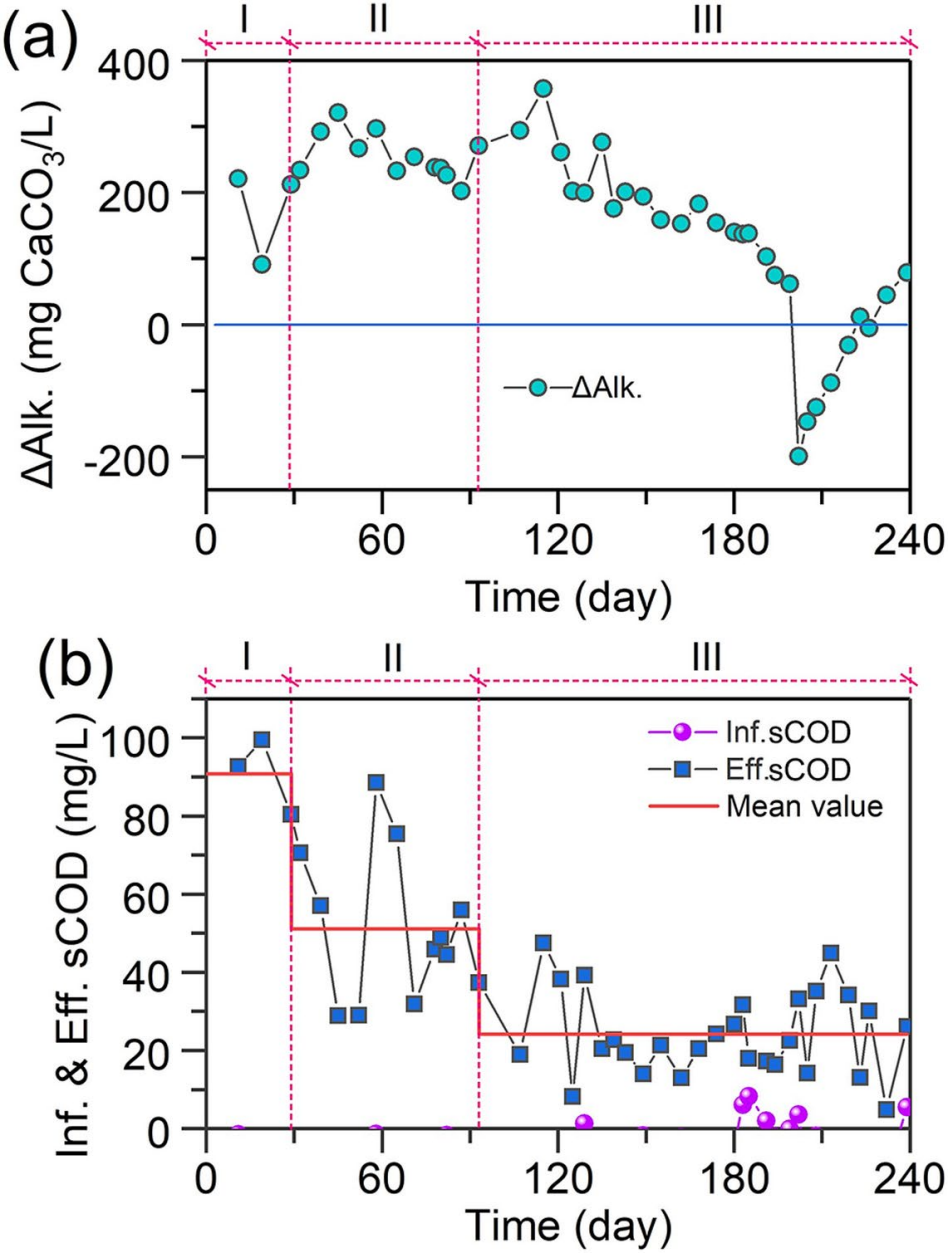
Temporal variations of **(a)** ΔAlk and **(b)** sCOD in IFBBR.

In order to explore the interactions between microalgae, nitrifiers and denitrifiers during 240 days of operation, alkalinity and sCOD were monitored. The temporal variations of ΔAlk. value, shown in Figure 3a, were used to evaluate the activity of nitrification, denitrification and microalgal ammonia assimilation in this work. Theoretically, nitrification consumes 7.14 g CaCO_3_/g NH_4_^+^-N_oxidized_ while denitrification generates 3.57 g CaCO_3_/g N_reduced_ (Tchobanoglous et al., 2014). In addition, microalgal ammonia assimilation consumes 3.57 CaCO_3_/g NH_4_^+^-N_assimilated_. During Phases I and II, the ΔAlk. remained above 200 mg CaCO_3_/L, suggesting that denitrification was the dominant nitrogen removal pathway. However, with the TN removal at approximately 100 mg/L in Phases I and II, the Alk. generated by denitrifiers should be around 357 mg CaCO_3_/L, greater than the real ΔAlk. value (approximately 260 mg CaCO_3_/L), this can be due to the presence of nitrifiers and microalgae which consumed the Alk. from day 121 to day 190, there was a sustained decline of ΔAlk., indicating that the increase of NLR led to a significant growth of nitrifiers and microalgae. Interestingly, as shown in Figure 2b, after day 121, TN removal efficiency kept increasing, suggesting the growth of denitrifiers. Results further indicated that with the growth of nitrifiers and microalgae, denitrifiers grew simultaneously.

Figure 3b shows the temporal variations of sCOD in influent and effluent during 240 days of operation. Except for a few instances (day 129, 183,185,191, 202) in which microalgae grew in the feed tank, the sCOD in influent remained at 0 throughout the rest of the time. Previous study found that the sCOD could be contributed by ammonia nitrogen (0.45 mg COD/ mg NH_4_^+^-N), and nitrite nitrogen (1.24 mg COD/mg NO_2_^−^-N), as shown in Supplementary Figure 1. However, even after subtracting the sCOD contributions from ammonia and nitrite nitrogen, the effluent still exhibited a relatively high sCOD level. Results indicate that organic carbon was generated inside the IFBBR, while some microorganisms such as microalgae could convert the inorganic carbon into organic carbon. Additionally, the effluent sCOD values in Phase III were lower than those in Phase II (26 ± 12 mg/L vs. 52 ± 21 mg/L), potentially due to the enhancement of denitrification as reflected by the aforementioned increase in TN removal efficiency.

The results above indicate that during the 240 days of operation, nitrification and denitrification occurred simultaneously due to the growth and accumulation of microalgae at the bottom wall of the IFBBR. Especially from day 121 to day 190, the notable improvement in ammonia nitrogen and TN removal performance revealed the enhancement of nitrification and denitrification. Generally, microalgae contribute to nitrogen removal in two ways: directly assimilating ammonia nitrogen (Gu et al., 2021; Lu et al., 2022) and synergistically increasing the activity of bacteria.

Microalgae provided the necessary O_2_ and organic carbon for nitrification and denitrification, while bacteria supplied CO_2_ to microalgae (Yan et al., 2022). In addition, microalgae can influence the composition and characteristics of EPS, with weak light entering through the observation window, both microalgae and bacteria grew on the smooth wall at the bottom of the IFBBR. In summary, enhanced nitrogen removal was first achieved in the IFBBR without external air and organic carbon source by coupling the SND with microalgal photoautotrophic assimilation.

### 3.2 Microbial activities in the IFBBR

**Figure 4:**
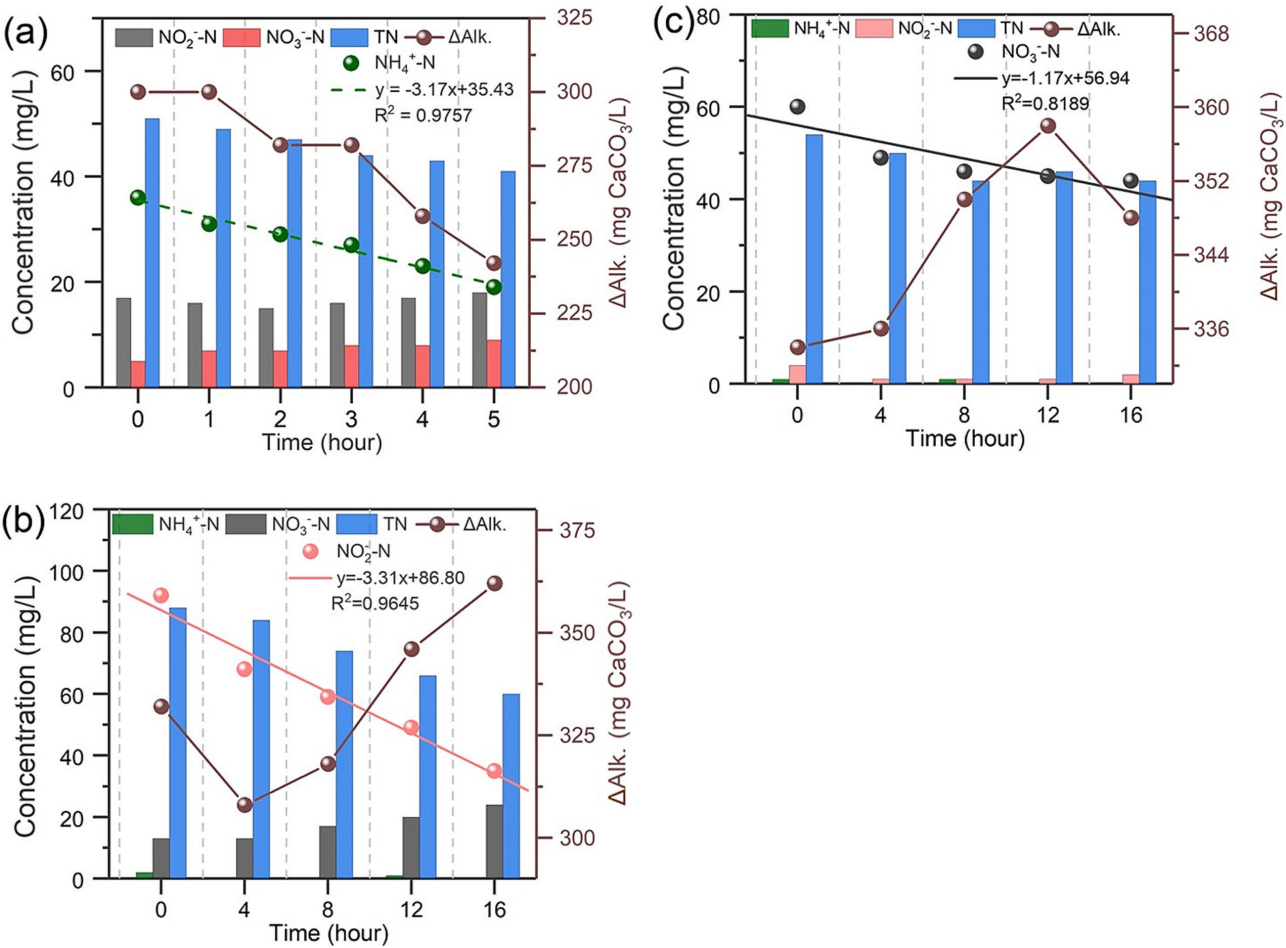
Nitrogen removal performance of IFBBR in Microbial Activities batch tests **(a)** First batch test (Ammonia); **(b)** Second batch test (Nitrite); **(c)** Third batch test (Nitrate).

The results of in-situ batch tests for nitrogen removal performance, shown in Figure 4, indicate the simultaneous presence of nitrifiers, denitrifiers and microalgae. Interestingly, the concentrations of ammonia, nitrite, and nitrate nitrogen showed a significant and sustained decline even when the IFBBR was purged by nitrogen gas and sealed after each sample collection. In addition, the only carbon-containing substance in the initial solutions was HCO_3_^−^. The O_2_ required for nitrification and the organic carbon required for denitrification were generated within the IFBBR, possibly due to the presence of microalgae. Moreover, as apparent from Figure 4, the SNRR of NH_4_^+^ -N, NO_2_^−^ -N, NO_3_^−^ -N, calculated from the slope of nitrogen concentration over time were 3.17, 3.31, and 1.17 mg/L·h, respectively. In addition, the SNRR of TN in the three batch tests were 2.03, 1.85, and 0.6 mg/L·h, respectively, indicating the nitrogen can be removal within the HRT of 48 h. In the third batch test, which focused on nitrate nitrogen removal, the NO_3_^−^ -N removal rate was significantly slower and most parameters remained stable in the final hours. This could be attributed to the DO accumulation in the absence of nitrification and the slow decay of microalgae and nitrifiers. The in-situ batch tests further confirmed the simultaneous occurrence of nitrification and denitrification within the IFBBR, in which SND was coupled with microalgal photoautotrophic assimilation. Microalgae generated the necessary O_2_ and organic carbon for nitrification and denitrification.

### 3.3 Microbial community analysis

#### 3.3.1 Distribution of eukaryotic community

**Figure 5:**
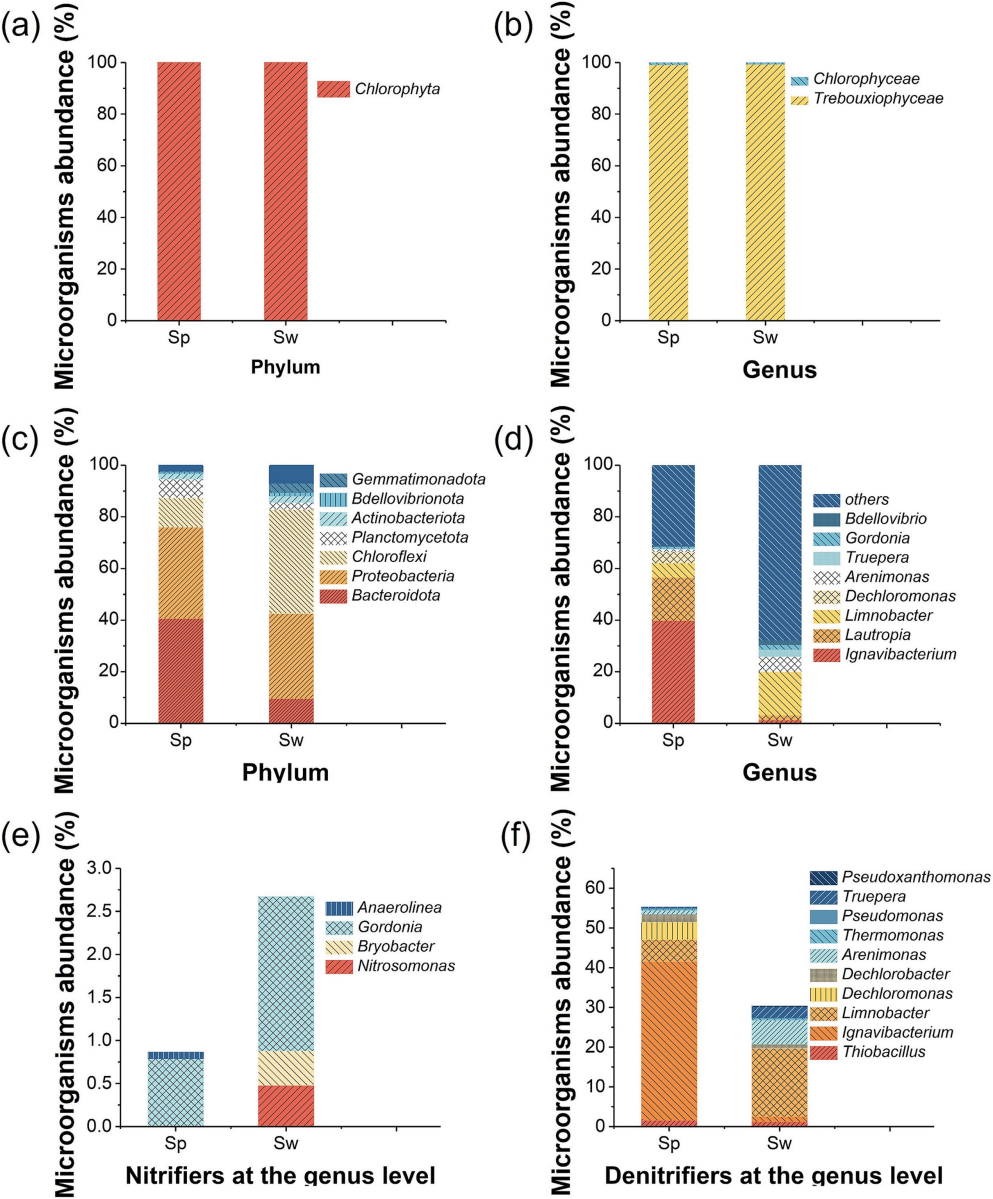
Relative abundance of microalgae and bacteria on particles (Sp) and the wall (Sw) in the IFBBR. **(a)** Eukaryotic community at phylum level; **(b)** Eukaryotic community at genus level; **(c)** Prokaryotic community at phylum level; **(d)** Prokaryotic community at genus level; **(e)** Nitrifiers at genus level; **(f)** Denitrifiers at genus level.

The analysis of the eukaryotic community (Figure 5b) indicated that *Trebouxiophyceae* was the dominant genus accounting for 99.04% of the total eukaryotic community in S_p_ and 99.26% in S_w_. *Trebouxiophyceae* is green microalgae that has high ammonium tolerance (Díaz et al., 2024). The remaining eukaryote belongs to the genus of *Chlorophyceae*. Both *Trebouxiophyceae* and *Chlorophyceae* belong to the phylum *Chlorophyta* (Figure 5a), which has the capability of autotrophic photosynthetic growth with inorganic substrates or heterotrophic growth by organic substrates assimilation (Jin et al., 2023).

The seed sludge used in the previous SBR was the TWAS, which was environmental sample that could contain bacteria and microalgae. During the 240 days of operation in the IFBBR, microalgae grew and accumulated in the IFBBR. Microalgae can influence the composition and characteristics of EPS (Jin et al., 2023), allowing biofilm to accumulate on the smooth surface of the wall. In the microalgal-bacterial symbiosis system, microalgae can capture CO_2_ dissolved in water or released by bacteria and then produce O_2_ for bacterial growth (Lu et al., 2022). Meanwhile, organic carbon synthesized by microalgae could serve as the electron donor for bacteria (Wang et al., 2015). Microalgae can also absorb growth-promoting substances by bacteria (Huang et al., 2022) and affect bacterial metabolism through signaling communication (Wu et al., 2024), which promotes the growth of both microalgae and bacteria.

#### 3.3.2 Distribution of prokaryotic communities

Figure 5c illustrates the prokaryotic community in two different areas (particle and wall) in the IFBBR at the phylum, the top three dominant phyla in S_p_ and S_w_ were identical, namely *Bacteroidota*, *Proteobacteria* and *Chloroflexi*. The relative abundance of *Bacteroidota* was 41% in S_p_ and 10% in S_w_, which is commonly found in WWTPs (Zhang H. et al., 2021). *Proteobacteria* had a high relative abundance in both S_p_ and S_w_, 35 and 33%, respectively. Many kinds of microorganisms belonging to *Proteobacteria* and *Bacteroidota* are related to the removal of nitrogenous compounds by denitrification (Yang et al., 2024; Zhang G. et al., 2024).

The relative abundance of *Chloroflexi* in S_w_ was higher than that in S_p_ (41% vs. 10%). *Chloroflexi* is typically a filamentous bacterium with extremely diverse nutritional methods including photoautotrophy, chemoautotrophy, photoheterotrophy, and chemoheterotrophy (Davis et al., 2011; Thiel et al., 2019). *Chloroflexi* is well-adapted to anaerobic environments and exhibits light orientation, serving as a bridge for sludge flocs and facilitating the formation of microalgal-bacterial symbiosis systems (Johnston et al., 2019). This may be the reason that the *Chloroflexi* abundance on the wall was more than that on the particle, and it could also confirm that an anoxic microenvironment existed in the IFBBR.

In addition, *Chloroflexi* is commonly found in BNR processes. Members of this phylum are also capable of inorganic CO_2_ fixation, aerobic nitrite oxidation, and nitrate reduction (Narsing Rao et al., 2022). Several publications have highlighted the significant role of *Chloroflexi* in autotrophic systems (Chu et al., 2015), in which they degrade complex compounds such as polysaccharides and proteins, and even utilize the decaying bacteria cell materials to generate energy through chlorophyll-mediated photosynthesis (Zhu et al., 2024).

Figure 5d presents the bacterial community diversity and richness at the genus level. The abundances of *Ignavibacterium* (40% vs. 1%), *Lautropia* (17% vs. 2%), *Dechloromonas* (4% vs. 0%) in S_p_ are much greater than those in S_w_. While the abundances of *Limnobacter* (6% vs. 17%), *Arenimonas* (1% vs. 6%), *Truepera* (3% vs. 0.2%) in S_p_ are much lower than those in S_w_. As can be seen in Figure 5e, even if the relative abundance of nitrifiers in S_p_ and S_w_ was low, AOB and NOB were present in both two areas. *Gordonia* (0.8% in S_p_ vs. 1.8% in S_w_) belongs to NOB (Zhang et al., 2023). Interestingly, some species of *Gordonia*, such as *polyisoprenivorans*, are capable of degrading organic pollutants (Wang et al., 2023). *Bryobacter* (0.0% in S_p_ vs. 0.4% in S_w_) and *Nitrosomonas* (0.0% in S_p_ vs. 0.5% in S_w_) which are AOB (Liu et al., 2024), were only present on the wall of the IFBBR. Moreover, another research indicated that *Anaerolinea* (0.1% in S_p_ vs. 0.0% in S_w_) was the primary AOB in the microbial electrolysis cells, used for wastewater nitrification (Yang et al., 2024). Overall, the relative abundance of AOB and NOB in S_w_ was higher than that in S_p_, which may be due to the microalgae on the wall generating O_2_ and nutrients, creating the necessary microaerobic environment and further promoting the growth of nitrifiers, as evidenced by the results in Section 3.1.

Figure 5f presents the relative abundance of denitrifiers at the genus level. *Ignavibacterium* (40.0%), *Limnobacter* (5.5%) and *Dechloromonas* (4.4%) were dominant in S_p_, while *Limnobacter* (17.1%), *Arenimonas* (6.0%) and *Truepera* (2.8%) were dominant in S_w_. *Ignavibacterium* and *Truepera* are common heterotrophic denitrifiers (Chen et al., 2024; Li et al., 2022). *Arenimonas* belongs to denitrifiers and Egbadon et al. (2024) suggested it was active in a microaerobic bioreactor. In addition, as shown in Figure 5d, *Lautropia*, a heterotrophic denitrifier (Sun et al., 2018; Zhuang et al., 2024), had a higher abundance in S_p_ than in S_w_ (16.9% vs. 1.9%). The different distribution of these bacteria may be due to varying microenvironments. Microalgae on the wall generated O_2_ which could inhibit the activity of a significant proportion of denitrifying bacteria. *Limnobacter* is a denitrifier with the capability of SO_4_^2−^ reduction (Wang et al., 2024). *Thiobacillus* (1.6% in S_p_ and 1.24% in S_w_) is known as a sulfur-based autotrophic denitrifying bacterium (Peng et al., 2021). By reducing SO_4_^2−^ in the nutrient solution, *Limnobacter* could supply reduced sulfur to *Thiobacillus*, facilitating sulfur-based autotrophic denitrification in the IFBBR.

Microbial community analysis further confirmed the existence of microalgae, nitrifiers and denitrifiers. Autotrophic nitrifiers and heterotrophic denitrifiers were the major nitrogen removal bacteria. O_2_ required for nitrification and organic carbon required for denitrification could be generated by microalgae. Meanwhile, anoxic/aerobic microenvironments were created, leading to the different distribution of nitrifiers and denitrifiers. In summary, microalgae played an important role in the IFBBR: preferentially assimilating ammonia nitrogen and promoting SND. Due to the coupling of SND with microalgal photoautotrophic assimilation, a novel microalgal-bacterial symbiosis system was first realized in the IFBBR without aeration and external organic carbon. This study might provide a new solution to achieve efficient nitrogen removal while reducing energy usage, operating cost, and CO_2_ release for WWTPs.

### 3.4 Results of C-N-O dynamic balance model

**Figure 6:**
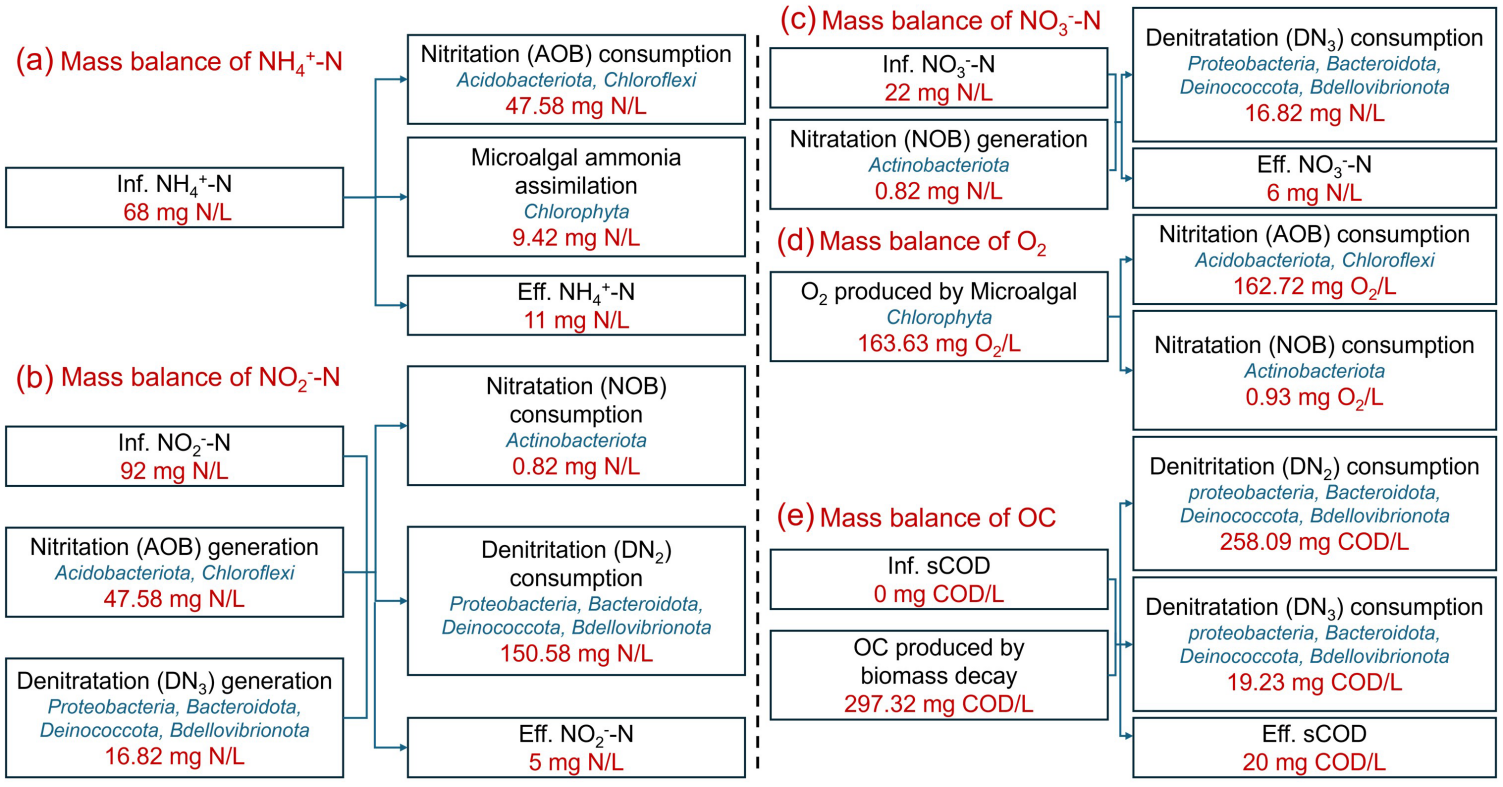
Mass balances of NH_4_^+^-N **(a)**, NO_2_^−^-N **(b)**, NO_3_^−^-N **(c)**, O_2_ **(d)**, and organic carbon (OC) **(e)** in IFBBR.

To elucidate the nitrogen removal pathway and the mechanisms of O_2_ and organic carbon generation, a C-N-O dynamic balance model was developed based on the reaction chemical formulae and theoretical parameters of microalgae and bacteria. In combination with the experimental water quality data (Supplementary Table 2), when the TN removal efficiency stabilized above 90%, the results of the C-N-O dynamic balance model were established, as shown in Figure 6. These results illustrate the mass balances of NH_4_^+^-N, NO_2_^−^-N, NO_3_^−^-N, O_2_ and organic carbon (OC).

As mentioned in Section 3.1 and further confirmed in Section 3.3, microalgae were present in the IFBBR. Figures 6a–c illustrate the mass balances of NH_4_^+^-N, NO_2_^−^-N, NO_3_^−^-N, respectively. In the IFBBR, there were two possible mechanisms for NH_4_^+^-N removal: nitritation (AOB, 47.58 mg N/L) and microalgae assimilation (9.42 mg N/L) (Zhang H. et al., 2021). Nitrite nitrogen could be generated by nitritation (AOB) and denitratation (DN_3_), while consumed by nitratation (NOB, 0.82 mg N/L) and denitritation (DN_2_, 150.58 mg N/L). Nitrate nitrogen could be generated by nitratation (NOB) and consumed by denitratation (DN_3_, 16.82 mg N/L). Figures 6d,e illustrate the mass balances of O_2_ and COD, respectively. O_2_ (163.63 mg/L) was generated by microalgae (Yan et al., 2022) and consumed by AOB (162.72 mg/L) and NOB (0.93 mg/L). COD was produced by microbial biomass decay (297.32 mg/L) and consumed by DN_2_ (258.09 mg COD/L) and DN_3_ (19.23 mg COD/L).

The C-N-O dynamic balance model further explained the interactions between microalgae, nitrification and denitrification in the IFBBR. The calculations and coefficients in the model were derived from alkalinity variation, reaction stoichiometry and theoretical parameters of microalgae nitrogen assimilation, nitrification and denitrification. Consequently, this model can be widely applied to various nitrification and denitrification reactors with or without the microalgae. However, for large-scale implementation and practical applications, the model parameters should be appropriately adjusted t depending on specific operational conditions.

## 4 Conclusion

To address the challenges of aeration and external organic carbon requirements in BNR process, this study successfully achieved enhanced nitrogen removal without aeration or external organic carbon. The innovative integrated IFBBR system, combining SND with photoautotrophic assimilation, was rapidly started up in 11 days and maintained stable operation for 240 days with weak light. During the stable period following the rapid startup, the TN removal efficiency of the IFBBR stabilized at 72 ± 2%. After the influent NLR increased from 0.07 to 0.09 kg N/m^3^·d on day 88, nitrogen removal performance was further enhanced since day 121, the ammonia nitrogen removal rate showed a continuous upward trend. On day 190, with an HRT of 48 h, the ammonia nitrogen removal rate approached 100%, achieving a TN removal rate as high as 95%. Microbial community analysis confirmed the presence of microalgae and identified autotrophic nitrifiers and heterotrophic denitrifiers as the primary bacteria groups responsible for nitrogen removal. Furthermore, a C-N-O dynamic balance model was developed to provide a quantitative understanding of the interactions between microalgae and bacteria. O_2_ required for nitrification was generated by microalgae at a rate of 81.82 mg/L·d, while organic carbon required for denitrification originated from microbial biomass decay at a rate of 148.66 mg/L·d. This study provides a novel efficient nitrogen removal technology to minimize energy consumption, operating cost and CO_2_ emissions in BNR processes. Future research should focus on the performance of nitrogen removal with photoautotrophic assimilation in a scale-up IFBBR for the real WWTPs.

## Data Availability

The original contributions presented in the study are included in the article/Supplementary material, further inquiries can be directed to the corresponding authors.
