# Supplementary material for: Microalgae-bacteria symbiosis enhanced nitrogen removal from wastewater in an inversed fluidized bed bioreactor: performance and microflora
Source: Front Microbiol. 2025 Apr 28;16:1591974. doi: 10.3389/fmicb.2025.1591974 (PMC12066685; doi:10.3389/fmicb.2025.1591974)
Supplement: Supplementary file 1 [file Data_Sheet_1.docx]

Supplementary Material

# Supplementary Data

## Supplementary Text 1. Details of mass balances calculation

A C-N-O dynamic balance model was constructed to further ensure the nitrogen removal mechanisms. In this microalgae-bacterial symbiotic system, NH_4_^+^-N can be removed by two different mechanisms: nitrification and microalgae assimilation (Zhang et al., 2021). Equation (1) and (2) show the NH_4_^+^-N removal by nitrification and microalgae. Here, NH_4_^+^-N removal includes contributions from both nitrification and microalgae assimilation, where the nitrifying bacteria oxidize NH_4_^+^-N into NO_2_^-^-N and subsequently into NO_3_^-^_-_N, while microalgae uptake NH_4_^+^-N directly for its growth and metabolism.

$\text{N}\text{H}_{\text{4}}^{\text{+}}\text{ + 2HC}\text{O}_{\text{3}}^{\text{-}}\text{+ 2}\text{O}_{\text{2}}\text{ }\text{→}\text{ }\text{N}\text{O}_{\text{3}}^{\text{-}}\text{+}\text{ }\text{3}\text{H}_{\text{2}}\text{O}\text{ }\text{+}\text{ }\text{2C}\text{O}_{\text{2}}$ (1)

$\text{N}\text{H}_{\text{4}}^{\text{+}}\text{ + 7.6}\text{C}\text{O}_{\text{2}}\text{ }\text{+ 17.7}\text{H}_{\text{2}}\text{O}\text{ → }\text{C}_{\text{7}\text{.6}}\text{H}_{\text{8}\text{.1}}\text{O}_{\text{2}\text{.5}}\text{N}\text{ }\text{+}\text{ }\text{7.6}\text{O}_{\text{2}}\text{ }\text{+}\text{ }\text{15.2}\text{H}_{\text{2}}\text{O}\text{ }\text{+}\text{ }\text{H}^{\text{+}}$ (2)

In this study, the main source of inorganic carbon was the HCO_3_^-^ in the feed, as shown in Equation (3), and the alkalinity consumed by microalgal ammonia assimilation could be calculated by Equation (4) (Tchobanoglous et al., 2014) according the microalgal ammonia assimilation (Equation (2)).The results showed that microalgae generated 3.57 mg CaCO_3_ of alkalinity for every 1 mg of ammonia nitrogen assimilated. HCO_3_^-^ served as the primary source of inorganic carbon, enabling microalgae to assimilate carbon for photosynthesis.

$\text{HC}\text{O}_{\text{3}}^{\text{-}}\text{ +}\text{ }\text{H}^{\text{+}}\text{ }\text{⇌}\text{ }\text{H}_{\text{2}}\text{C}\text{O}_{\text{3}}^{\text{*}}$ (3)

$\text{Alk., mg/L as CaC}\text{O}_{\text{3}}\text{ = \{[HC}\text{O}_{\text{3}}^{\text{-}}\text{] + 2[C}\text{O}_{\text{3}}^{\text{2-}}\text{] + [O}\text{H}^{\text{-}}\text{] – [}\text{H}^{\text{-}}\text{] meq/L\} × }\frac{\text{50 mg CaC}\text{O}_{\text{3}}}{\text{meq CaC}\text{O}_{\text{3}}}$ (4)

There were two possible pathways for soluble nitrogen (sN) removal (Tang et al., 2018): denitrification and microalgal assimilation. Nitrite nitrogen could be generated by nitritation (AOB) (Equation (1)) and denitratation (DN_3_) (Equation (5)), consumed by nitratation (NOB) (Equation (7)) and denitritation (DN_2_) (Equation (6)). Nitrate nitrogen could be generated by nitratation (NOB) while consumed by denitratation (DN_3_).

$\text{N}\text{O}_{\text{3}}^{\text{-}}\text{ }\text{+ 2}\text{H}^{\text{+}}\text{ }\text{+ }\text{e}^{\text{-}}\text{ → N}\text{O}_{\text{2}}^{\text{-}}\text{ + }\text{H}_{\text{2}}\text{O}$ (5)

$\text{N}\text{O}_{\text{2}}^{\text{-}}\text{ + }\text{H}^{\text{+}}\text{+ }\text{e}^{\text{-}}\text{→ NO}\text{ }\text{+ }\text{H}_{\text{2}}\text{O}$ (6)

$\text{N}\text{O}_{\text{2}}^{\text{-}}\text{ }\text{+ 0.5}\text{O}_{\text{2}}\text{ → }\text{N}\text{O}_{\text{3}}^{\text{-}}$ (7)

The organic carbon could be generated by biomass decay and consumed by denitrification. Generally, the value of biomass decay rate (b_20_) is 0.12 g VSS/g VSS_total_ d (20 ℃), of which 90% (85 - 90%) is biodegradable (Tchobanoglous et al., 2014). With the increase of temperature (38 ℃), the biomass decay rate (b_r_) could also increase (as shown in Equation (8), Where *θ* is temperature activity coefficient, values for *θ* in biological systems can vary from 1.02 to 1.25.

$\text{b}_{\text{r}}\text{ = }\text{b}_{\text{20}}\text{ × (}\text{θ}^{\text{T-20}}\text{)}$ (8)

Theoretically, Alk. is consumed at 7.14 g/g NH_4_^+^-N_oxidized_ during nitrification and generated at 3.57 g/g NO_x_^-^-N reduced to N_2_ during denitrification (Tchobanoglous et al., 2014). In addition, this system was stable with the existence of microalgae, nitrifiers and denitrifiers, indicating that the oxygen produced by microalgae was consumed by nitrifying bacteria without accumulation.

The following seven COD, nitrogen, oxygen and alkalinity balance equations (Equation (9) - (15)) were used for the mass balance of phase III (day 155 - 174) when TN removal efficiency was stable at over 90% (data summarized in Supplementary Table 2.). Alkalinity (Alk.) plays a crucial role in balancing chemical processes, being consumed during nitrification and generated during denitrification and microalgal ammonia assimilation.

$\text{∆}\text{N}\text{H}_{\text{4}}^{\text{+}}\text{-N}\text{ }\text{= -N}\text{H}_{\text{4}}^{\text{+}}\text{-}\text{N}_{\text{AOB consumption}}\text{ - N}\text{H}_{\text{4}}^{\text{+}}\text{-}\text{N}_{\text{MA consumption}}\text{ = -57 mg/L}$ (9)

$\text{∆}\text{N}\text{O}_{\text{2}}^{\text{-}}\text{-N = N}\text{H}_{\text{4}}^{\text{+}}\text{-}\text{N}_{\text{AOB consumption}}\text{ - N}\text{O}_{\text{2}}^{\text{-}}\text{-}\text{N}_{\text{NOB consumption}}\text{ - N}\text{O}_{\text{2}}^{\text{-}}\text{-}\text{N}_{\text{DN2 consumption}}\text{ + N}\text{O}_{\text{3}}^{\text{-}}\text{-}\text{N}_{\text{DN3 consumption}}\text{ = -87 mg/L}$ (10)

$\text{∆}\text{N}\text{O}_{\text{3}}^{\text{-}}\text{-N = N}\text{O}_{\text{2}}^{\text{-}}\text{-}\text{N}_{\text{NOB consumption}}\text{ - N}\text{O}_{\text{3}}^{\text{-}}\text{-}\text{N}_{\text{DN3 consumption}}\text{ = -16 mg/L}$ (11)

$\text{∆sN = -}\text{N}\text{O}_{\text{2}}^{\text{-}}\text{-}\text{N}_{\text{DN2 consumption}}\text{ - N}\text{H}_{\text{4}}^{\text{+}}\text{-}\text{N}_{\text{MA consumption}}\text{ = -161 mg/L}$ (12)

$\text{∆Alk.}\text{ }\text{=}\text{ }\text{3.57 × }\text{N}\text{O}_{\text{2}}^{\text{-}}\text{-}\text{N}_{\text{DN2 consumption}}\text{ - 7.14 × N}\text{H}_{\text{4}}^{\text{+}}\text{-}\text{N}_{\text{AOB consumption}} \text{-}\text{ }\text{3.57 × N}\text{H}_{\text{4}}^{\text{+}}\text{-}\text{N}_{\text{MA consumption}}\text{ =}\text{ }\text{162 mg/L}$ (13)

$\text{∆}\text{O}_{\text{2}}\text{ =}\text{ }\text{17.37 × }\text{N}\text{H}_{\text{4}}^{\text{+}}\text{-}\text{N}_{\text{MA consumption}}\text{ -}\text{ }\text{1.14 × N}\text{O}_{\text{2}}^{\text{-}}\text{-}\text{N}_{\text{NOB consumption}}\text{ -}\text{ }\text{3.42 × N}\text{H}_{\text{4}}^{\text{+}}\text{-}\text{N}_{\text{AOB consumption}}\text{ = 0 mg/L}$ (14)

$\text{∆COD =}\text{ }\text{0.12 × }\text{θ}^{\text{18}}\text{ ×0.90 ×143 }\text{- 1.143 × N}\text{O}_{\text{3}}^{\text{-}}\text{-}\text{N}_{\text{DN3 consumption}}\text{ - 1.714 × N}\text{O}_{\text{2}}^{\text{-}}\text{-}\text{N}_{\text{DN2 consumption}}\text{ = 20 mg/L}$ (15)

As $\text{θ}$ values change, different nitrogen and alkalinity consumed concentrations were calculated, as shown in Supplementary Figure 2., in which it is reasonable for $\text{θ}$ values between 1.07 and 1.10. In summary, in this microalgal-bacterial system, oxygen was generated for nitrification by microalgae and organic carbon was generated for denitrification through biomass decay. Generally, NOB requires a higher DO concentration than AOB for its growth (Liu et al., 2020). The IFBBR and the synthetic wastewater were purged by pure nitrogen gas daily to reduce DO concentration and avoid interference from external oxygen, which could inhibit the nitratation. The reaction occurring at a θ value of 1.07 represents the actual conditions and the mass balance is presented inFigure 6 of the manuscript.

**Reference**

Liu, X., Kim, M., Nakhla, G., Andalib, M., & Fang, Y. (2020). Partial nitrification-reactor configurations, and operational conditions: Performance analysis. *J. Environ. Chem.* 8(4):103984. [doi: 10.1016/j.jece.2020.103984](https://doi.org/10.1016/j.jece.2020.103984)

Tang, C. C., Tian, Y., He, Z. W., Zuo, W., & Zhang, J. (2018). Performance and mechanism of a novel algal-bacterial symbiosis system based on sequencing batch suspended biofilm reactor treating domestic wastewater. *Bioresour. Technol.* 265:422-431. [doi: 10.1016/j.biortech.2018.06.033](https://doi.org/10.1016/j.biortech.2018.06.033)

Tchobanoglous, G., Stensel, H. D., Tsuchihashi, R., & Burton, F. (2014). Wastewater engineering: Treatment and resource recovery. Fifth ed., McGraw-Hill Education, New York.

Zhang, F., Peng, Y., Wang, Z., Jiang, H., Ren, S., & Qiu, J. (2021). Achieving synergetic treatment of sludge supernatant, waste activated sludge and secondary effluent for wastewater treatment plants (WWTPs) sustainable development. *Bioresour. Technol.* 337:125416. [doi: 10.1016/j.biortech.2021.125416](https://doi.org/10.1016/j.biortech.2021.125416)

# Supplementary Figures and Tables

## Supplementary Figures


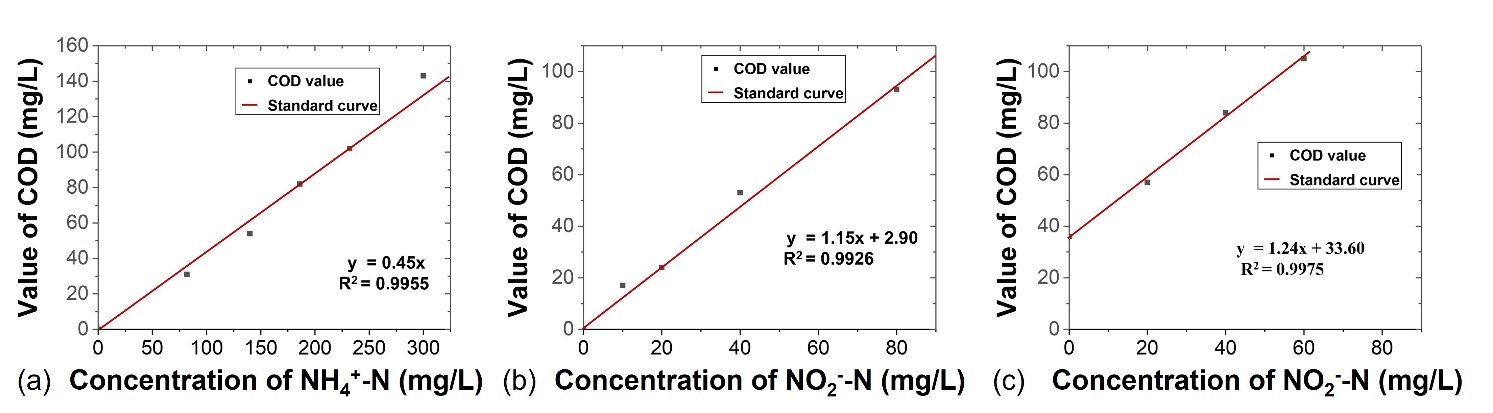


**Supplementary Figure 1.** Standard curves of nitrogen contribute to COD value in COD detection. (a) Contribution of Ammonia nitrogen to COD value; (b) Contribution of Nitrite nitrogen to COD value; (c) Contribution of nitrite nitrogen to COD value under the coexistence of nitrite and glucose.


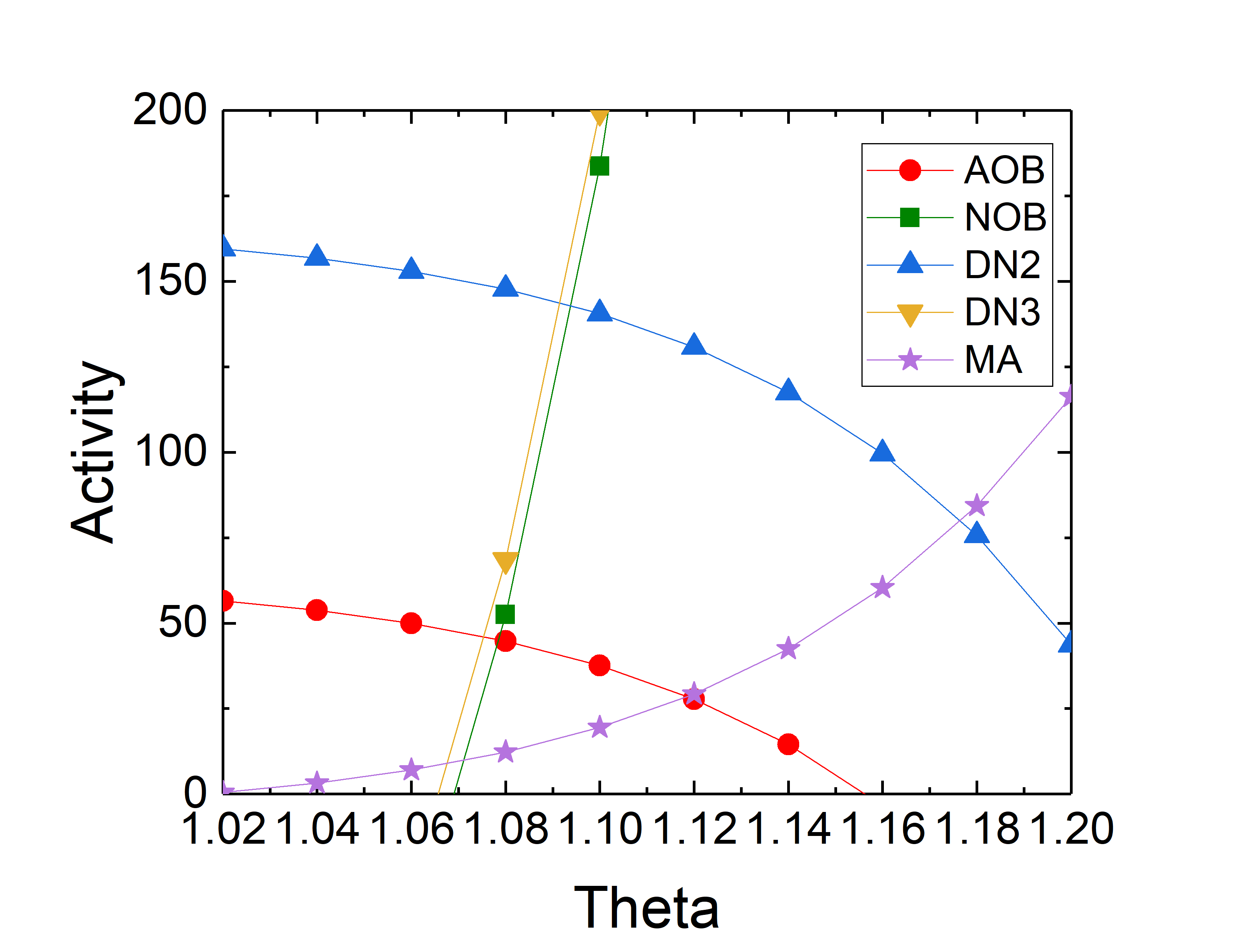


**Supplementary Figure 2.** Concentration of nitrogen and alkalinity consumed by AOB, NOB, DN2, and DN3 under different "θ" values.

## Supplementary Tables

**Supplementary Table 1.** Synthetic wastewater composition

| **Constituents of the synthetic medium** | **Concentration** |
| --- | --- |
| KH_2_PO_4_ | 27 mg/L |
| MgSO_4_ | 300 mg/L |
| CaCl_2_ | 100 mg/L |
| **Trace elements solution I** | **Concentration** |
| EDTA | 5 mg/L |
| FeSO_4_·7H_2_O | 5 mg/L |
| **Trace elements solution II** | **Concentration** |
| EDTA | 15 mg/L |
| CuSO_4_·5H_2_O | 0.25 mg/L |
| ZnSO_4_·7H_2_O | 0.43 mg/L |
| CoCl_2_·6H_2_O | 0.24 mg/L |
| MnCl_2_·4H_2_O | 0.99 mg/L |
| H_3_BO_3_ | 0.014 mg/L |
| NiCl_2_ | 0.1 mg/L |
| Na_2_MoO_4_·2H_2_O | 0.22 mg/L |
| Na_2_SeO_3_ | 0.1 mg/L |

**Supplementary Table 2.** The nitrogen removal and effluent water quality of IFBBR from day 155 to day 174 (Calculated by average).

|  | NH_4_^+^-N Removal | NO_2_^-^-N Removal | NO_3_^-^-N Removal | TN  Removal | ΔAlk. | Eff. VSS | Eff. pCOD | Eff. sCOD |
| --- | --- | --- | --- | --- | --- | --- | --- | --- |
| Day  155-174 | 57 mg/L | 87 mg/L | 16 mg/L | 161 mg/L | 162 mg CaCO_3_/L | 27 mg/L | 40 mg/L | 20 mg/L |
